
JOURNAL INFORMATION
==============================
NLM Title Abbreviation: Buffalo Med J
NLM Journal ID: 0027534

Buffalo Medical Journal

ISSN: 1040-3817
Publisher: Biomedical Journal Digitization Project

ARTICLE INFORMATION
==============================
PMCID: PMC8735419
PMID: 36886866
Article version: 1
Subjects: Items

Items

Print publication date: 1906 Apr
Volume: 61
Issue: 9
First page: 570
Last page: 570
License: This work is free of known copyright restrictions. For more information, please see PMC Back Issue Digitization.
License URL: https://creativecommons.org/publicdomain/mark/1.0/

==============================

---

The United States Civil Service Commission announces an examination on June 6-7, 1906, at the usual places, to secure eligibles from which to make certification to fill at least two vacancies, at $600 per annum each, with maintainence, in the position of medical interne, Government Hospital for the Insane, Washington, D. C., and vacancies as they may occur in any branch of the service requiring similar qualifications.

The Department states that it reserves the right to continue or terminate appointment at the end of one year, or to promote the appointee at the expiration of that length of service.

### ITEMS.

---

The second hand, Harvard operating chairs advertised for sale in this issue by Mr. L. D. Platt, No. 1050 Ellicott Spuare, are as good as new. Mr. Platt is the Buffalo representative of the W. D. Allison concern of Indianapolis, and of the Wagner Electrical Co., of Chicago.

---

Mr. Wm. T. Collins, whose card appears in the Journal, has been in the medical book business in Buffalo for the past thirty years. He can obtain any medical book published, on short notice. See him for medical books.

---

**WANTED.**—Copies of the **BUFFALO MEDICAL JOURNAL** for the months of **AUGUST** and **DECEMBER**, 1905, for which the full price of twenty-five cents each will be paid, delivered to the Editor, 284 Franklin Street, Buffalo.
